# *Mycobacterium tuberculosis* as a model system

**DOI:** 10.1128/jb.00609-25

**Published:** 2026-03-16

**Authors:** Emilee Barnard, M. Maria Elgrail, Allison Fay, Yaprak Ozakman, Michael S. Glickman

**Affiliations:** 1 Immunology Program, Memorial Sloan Kettering Cancer Center, Sloan Kettering Institute 132189 , New York, New York, USA; 2 BCMB Graduate Program, Weill Cornell Medicine 12295, New York, New York, USA; Dartmouth College Geisel School of Medicine, Hanover, New Hampshire, USA

**Keywords:** mycobacterium, tuberculosis

## Abstract

Over the past 30 years, the study of *Mycobacterium tuberculosis* has become a vibrant field that supports basic investigation into mycobacteria, wide-ranging studies of *M. tuberculosis* pathogenesis, and drug and therapeutic development. In this minireview, we highlight the multiple ways in which *M. tuberculosis* is a model. *M. tuberculosis* has emerged as a model for prokaryotic biology, revealing basic mechanisms of gene expression, genomic integrity, and mutagenesis that expand prokaryotic dogmas established in earlier model organisms. Next, we highlight the experimental systems used to model *M. tuberculosis* human infection. As an obligate human pathogen, all attempts to understand the pathogenesis of TB disease seek to mimic the human infection, and a variety of systems have emerged from our field. Finally, we highlight that the *M. tuberculosis* field is a model for a community-driven scientific approach to a pressing global health problem for which commercial interest is limited. Our field has taken on the challenge of drug and diagnostic development, based on a foundation of expanding knowledge of basic *M. tuberculosis* biology, by working through academic and public/private partnerships, to make meaningful progress in therapeutics and diagnostics. Altogether, we present the *M. tuberculosis* field as a model for the development of a productive community that spans basic, translational, and therapeutic research, supported by public and philanthropic funding, to ultimately positively impact the treatment of human TB and the advancement of our knowledge base of microbiology and microbial pathogenesis.

## WHAT DOES IT MEAN TO TALK ABOUT *MYCOBACTERIUM TUBERCULOSIS* AS A MODEL SYSTEM?

We are thrilled to contribute an installment in this series on microbial model systems to the *Journal of Bacteriology*. In launching this series (1), the editors envisioned that each installment could serve as an introduction to a field for the major bacterial model systems. In this sense, writing this installment on *Mycobacterium tuberculosis* (Mtb) presents some great opportunities and some challenges. There are already two excellent installments in this series covering *Mycobacterium smegmatis* (2) and *Mycobacterium marinum* (3), and we refer the reader to these reviews for related insights on the use of these mycobacteria as models. The most substantive challenge is to define how *M. tuberculosis* is a model, and what is it a model for? In considering these challenges, we have chosen to highlight several different interpretations of “*M. tuberculosis* as model.” We first consider *M. tuberculosis* as a model system for prokaryotic biology and emphasize the areas for which the study of Mtb has expanded prokaryotic dogmas established from the earlier model systems of *Escherichia coli* and *Bacillus subtilis*. Second, we consider experimental systems that seek to model the pathogenesis of tuberculosis as a human disease. As an obligate human pathogen, all attempts to use surrogate systems are ultimately imperfect models of the human infection. Finally, we highlight the ways in which *M. tuberculosis,* as a global pathogen with massive disease burden but little commercial interest in therapeutic development, has been a model for public-private partnerships in drug and diagnostic development, with notable successes that can be a model for other fields. Common to all of these interpretations of “Mtb as model” are two inescapable microbiologic features that impact all experimental work with Mtb: its slow growth rate and its requirement for BSL3 containment. Due to the nature of the broad scope of biology covered in this review, we apologize for our inability to cite all the excellent work that our field has contributed to the bacteriology literature over the past decades.

## *M. TUBERCULOSIS* AS A MODEL SYSTEM FOR PROKARYOTIC BIOLOGY

### DNA repair and mutagenesis

The paradigmatic models of DNA repair emerged from extensive studies in *E. coli* and *B. subtilis* that later served as the model for studies in eukaryotic cells (4–6). The foundational principles governing homologous recombination, regulation of the DNA damage response (DDR), mismatch repair (MMR), and many other DNA repair pathways were established through studies in *E. coli* (4, 5, 7–10). However, extrapolating these principles to all bacteria underestimates the diversity of prokaryotic repair strategies. *M. tuberculosis* and related mycobacteria have served as a fertile system to study new DNA repair and mutagenesis pathways absent in these earlier model systems. In prokaryotes, GC content can range from ~20% to ~75% (11), and this variation necessitates the identification of representative model organisms with the same GC range. The GC content of *E. coli* is approximately 50%, and in *B. subtilis,* it is approximately 43%, yet in Mtb, it is ~66% (12). Representation of GC-rich species rises in importance in light of arguments from evolutionary genomic studies that there may be selection to increase synonymous GC content in many bacterial species (13) and the possibility that GC content might influence the mechanisms and outcome of DNA repair systems.

### Double-strand break repair

RecA-dependent homologous recombination is the dominant pathway of double-strand break (DSB) repair in most bacteria. In contrast, eukaryotes express multiple DSB repair pathways, including nonhomologous end-joining, which can directly repair DSBs without a homologous template through the action of the DNA end-binding protein Ku and ATP-dependent DNA ligases. NHEJ was long thought absent from bacteria, but the presence of genes encoding Ku proteins in bacterial genomes (14) and the sensitivity of *B. subtilis* spores lacking Ku or LigD to ionizing radiation (15) suggested a prokaryotic NHEJ pathway. Mycobacteria have been the primary system used to elucidate the functional characteristics of bacterial NHEJ, which is also present in *Pseudomonas* and other bacteria. Indeed, a 2016 search through 2,645 prokaryotic complete genomes suggested that ~25% possessed genes encoding for putative Ku proteins, with particular representation among Actinobacteria, Firmicutes, Proteobacteria, and Bacteroidetes phyla (16). Mycobacterial studies have revealed a DSB repair system which is active in nonreplicating mycobacterial cells and that can modify the broken ends through the concerted action of the multifunctional LigD protein, with additional factors (17–23). It was proposed that this system may be useful in eluding genotoxins from the host, including nitric oxide and hydrogen peroxide, and that imperfect NHEJ may serve as an adaptive means for sequence diversification and survival in the nonreplicating state adopted by *M. tuberculosis* during clinical latency. Although an attractive hypothesis, a role for *M. tuberculosis* NHEJ in infection has not been demonstrated, possibly due to our inability to model the nonreplicating latent state in animal models (24). Mycobacteria elaborate yet another DSB repair system previously unknown in prokaryotes, a RecA-independent single-strand annealing pathway that relies on the RecBCD resection nuclease (which is dedicated by RecA-dependent homologous recombination in *E. coli*) and which repairs DSBs between repeats with deletion of the intervening sequence (22).

### Pathways and control of mutagenesis

The control of mutagenesis is of interest to many biological fields, including antibiotic resistance, cancer, and evolution (25, 26). In *M. tuberculosis*, prevention of chromosomal mutagenesis of its GC-rich genome must be maintained during constant exposure to environmental clastogenic stress and during prolonged nonreplicating states. Because all antimicrobial resistance mutations in Mtb are chromosomal and not the result of horizontal gene transfer, understanding the pathways of mutagenesis in Mtb is critical to understand, and potentially prevent, antimicrobial resistance (27, 28). In contrast to *E. coli* and many bacteria, in which the SOS response, mediated by RecA-mediated cleavage of the LexA repressor, controls DNA damage induced gene expression, *M. tuberculosis* has a dual control system. Early observations of the Mtb DNA damage response indicated that some damage-inducible transcription did not depend on RecA, which suggested a LexA-independent mechanism of activation operating in parallel to the classical LexA-repressed SOS response (29). Later studies identified the PafBC heterodimer as an activator of a large number of damage-inducible genes (30) through single-strand DNA binding (31). Resistance to DNA damage is co-dependent on LexA and PafBC (32), and mycobacterial RecA is subject to posttranslational regulation that modifies its activity in the SOS response (33, 34), adding a layer of complexity to our understanding of the bacterial DDR.

In the macrophage, Mtb can survive amidst an abundance of reactive oxygen species (ROS), which can damage proteins, lipids, and nucleic acids. Guanine is especially susceptible to oxidative damage (35), and it has been argued that the high GC content of the Mtb genome may be a vulnerability when managing the effects of ROS. Mtb has evolved multiple mechanisms for detoxifying ROS, including multiple antioxidant enzymes. When these defense systems fail, Mtb has mechanisms that prevent or repair the ROS-inflicted DNA damage, including frequent oxidation of guanine to 8-oxo-G, which is mutagenic through base mispairing. Mtb has multiple MutT proteins and two MutMs, and in *M. smegmatis*, MutM/MutY is the dominant system protecting against oxo-G-associated mutagenesis (36). Recent evidence suggests that some mycobacterial MutT enzymes also have divergent functions from their roles in oxo-G processing, including in RNA metabolism and protein dephosphorylation (37–39), providing yet another example of the divergence of *M. tuberculosis* biology from classical model organisms.

*M. tuberculosis* expresses a suite of translesion polymerases with roles in mutagenesis. The DNA polymerase DnaE2 is upregulated upon exposure to DNA-damaging agents, including ROS (40, 41), and is a major mediator of mutagenesis in Mtb. It has been proposed that DnaE2 confers a survival advantage via inducible mutagenesis, which increases opportunities for gaining drug resistance mutations (40, 42). It has also become clear that missense mutations, although critical for drug resistance and other phenotypes, are not the only source of sequence diversification in the *M. tuberculosis* chromosome. Insertions and deletions in homopolymeric tracts of identical nucleotides are increasingly recognized as a functionally important class of genome diversity in *M. tuberculosis* (43–45), which can modulate antibiotic sensitivity and virulence. Recent evidence also implicates *M. tuberculosis* translesion polymerases, which have vigorous frameshifting activity on homopolymeric tracts, in generating indels (42, 46–48).

In terms of other DNA repair systems, MMR is a pathway that recognizes and repairs mismatched nucleotides, and it displays strong conservation across many prokaryotic and eukaryotic species (49). Mutations in the crucial MMR genes *mutS* and *mutL* are frequently implicated in mutator phenotypes, including in both clinical and laboratory bacterial strains (25). However, homologs of canonical MMR genes are notably absent in Mtb (50), yet genomic mutation rates in wild-type Mtb do not differ dramatically from those of prokaryotes that do have MMR (51). These observations led to the identification of a new class of mismatch repair systems mediated by the NucS endonuclease (52), that have now been identified and studied in non-mycobacterial clades and Archaea (53, 54).

## *M. TUBERCULOSIS* AS A MODEL FOR PROKARYOTIC TRANSCRIPTION

The study of bacterial gene expression has long been guided by principles established in *E. coli* and *Thermus aquaticus*, where transcription is carried out by a conserved RNA polymerase (RNAP) holoenzyme and directed by a set of sigma (σ) factors (55–57). However, emerging mechanistic studies performed with Mtb RNAP challenge the assumption that these paradigms are universal to bacteria (57–62). Despite broad conservation of RNAP structure, Mtb exhibits distinctive transcriptional features that influence promoter selectivity, open complex stability, and regulatory logic. These differences, combined with its slow growth rate and adaptation to host environments, position Mtb as a compelling model system for understanding transcriptional adaptation in host environments, particularly in the context of persistence and host-pathogen interactions.

### Distinctive features of mycobacterial RNA polymerase

While the core RNAP structure in Mtb shares the same subunit composition common to most bacteria (αββ′⍵), subtle yet significant structural distinctions exist, especially in the β and β′ subunits, which are encoded by *rpoB* and *rpoC*, respectively (61). Most notably, the β subunit is the primary target of rifamycins, a class of first-line anti-mycobacterial drugs such as rifampin (60). Most rifampin resistance-conferring mutations map to a defined region of RpoB termed the rifampin resistance-determining region. Structural analyses of Mtb RNAP have shown how RpoB mutations, such as Ser450Leu, block rifampin binding, leading to altered transcription initiation kinetics and significant fitness costs (63, 64). These insights are critical for understanding the fitness landscape of resistance mutations and for the development of drugs with high genetic barriers to resistance.

Beyond drug-target interactions, Mtb transcription diverges from other bacterial models through the reliance of Mtb RNAP on a distinct set of essential general transcription factors, such as RbpA and CarD (62, 65). Both factors interact directly with RNAP and stabilize the RNAP open promoter complex (RPo) (61). Whereas *E. coli* RNAP forms an essentially irreversible open complex on most promoters and nearly always transitions to elongation, Mtb RPo is inherently unstable and dissociates from promoter DNA without these factors. CarD binds the upstream edge of the transcription bubble, while RbpA contacts σ factor and the RNAP β subunit, cooperatively enhancing RPo stability (66–71). This additional layer of regulation likely compensates for intrinsically weak mycobacterial promoters and may represent an adaptive feature that enables rapid transcriptional modulation under stress.

### Sigma factor networks and transcriptional regulation in *M. tuberculosis*

Another substantial divergence of Mtb RNAP from other model bacteria is in the regulatory capacity conferred by the σ factor repertoire. Mtb encodes at least 13 sigmas, including the essential primary factor σ^A^ and 10 extracytoplasmic function (ECF) σ factors (72–74). This diversity of regulators, which is shared with other pathogenic and nonpathogenic bacteria that contend with diverse environments (75), confers Mtb with significant regulatory flexibility, which is essential for infection and intracellular persistence.

σ^A^ serves as the principal housekeeping factor (76) which is functionally analogous to *E. coli* σ^70^, directing transcription of genes required for growth (53). In contrast, the alternative σ factors, primarily from the ECF family, mediate specific stress responses and operate in a tightly integrated network (77, 78). For example, σ^E^ and σ^H^ are central to the oxidative and envelope stress responses, controlling genes involved in cell envelope integrity and protein quality control (79, 80). σ^F^ is a key regulator for the switch to nonreplicating persistence, often induced by nutrient limitation (81, 82). The closely related σ^A^-like factor, σ^B^, is also activated during stress-induced transitions and enhances general stress resistance. Other ECF factors, like σ^L^ and σ^M^, regulate pathways for lipid metabolism and nutrient limitation (73). Many Mtb strains lacking ECF sigma factors exhibit mild phenotypes in mouse infection models, but in at least one case, alternative infection hosts revealed an important function: non-human primates (NHPs) infected with a Mtb ∆*sigH* strain did not develop acute tuberculosis, a much more severe phenotype than in mice (83). Regulatory complexity in Mtb also arises from its extensive network of anti-σ factors. Nearly every non-housekeeping σ factor in Mtb has a cognate anti-σ factor that sequesters it until an environmental cue, such as oxidative, envelope, or heat stress, triggers release via proteolysis or conformational change (77, 84–86). This mechanism allows fast, modular transcriptional reprogramming, as σ-anti-σ pairs function as both direct sensors and transducers of stress.

While some of these features, such as reliance on σ factors and open complex formation, are conserved across bacteria, the combination of unstable open complexes, stabilizing essential general RNAP factors, and an expanded σ factor network is unique. For example, *E. coli* relies heavily on DNA-binding transcription factors to achieve regulatory diversity, whereas Mtb places comparatively more regulatory weight on σ factor regulation through an expanded σ factor repertoire and anti-σ factor control underpinning a regulatory logic that is central to Mtb pathogenicity and drug tolerance (72, 87–89). This expanded sigma factor network works in concert with similarly expansive regulatory systems of two-component systems and serine-threonine protein kinases (90–92). Collectively, these unique features and regulatory complexities position Mtb as an informative model for understanding bacterial transcription, particularly in the context of adaptation to challenging environments.

## *M. TUBERCULOSIS* AS A MODEL FOR PATHOGENIC MECHANISMS AND HUMAN TB

As a pathogen, *M. tuberculosis* was a latecomer to the field of bacterial pathogenesis. Despite its undeniable global disease burden, detailed mechanistic experiments in Mtb were difficult, due to technical limitations and the aforementioned slow growth and requirement for biocontainment. The earliest model systems for bacterial pathogenesis were those for which genetic systems were available: Gram negatives such as *E. coli*, Salmonella, and Shigella, and *Listeria monocytogenes*. Pathogenic mycobacteria were genetically intractable and therefore did not join the ranks of microbial pathogen model systems until genetic systems were developed. We will not review the early studies that led to genetic tractability of mycobacteria, as these are covered in Sparks et al. (2), but the history of Mtb as a microbial model system is approaching 25 years (93), and dramatic advances have been achieved in our understanding of this pathogen.

The study of Mtb pathogenesis requires cellular or animal models, which would ideally fully recapitulate the unique biology of within the natural and obligate human host. *M. tuberculosis* is a human pathogen without a known environmental reservoir. Infection is initiated by inhalation of aerosolized bacteria expelled by the cough of an active pulmonary TB case. Inhaled bacteria deposit in the lung, and, after a period of initial replication, which is asymptomatic in the vast majority of immunocompetent adults, the bacterium is reduced to a state of clinical dormancy which continues to be asymptomatic, and bacteria are not cultivatable. Although there has been recent debate about the duration, universality, and terminology applied to this period after TB exposure (94, 95), the end of this post-exposure period, which can last years, is (most commonly) pulmonary TB. The pathologic hallmark of human tuberculosis is the granuloma, a stereotypic pattern of inflammation characterized by infected macrophages at its core, surrounded by a rim of lymphocytes. Although not specific for TB infection, this immunologic structure is a critical feature of the immune response to TB, and significant efforts continue to understand the pathologic and protective features of granulomas. Any model of TB infection would ideally recapitulate many of these features: aerosol infection, an asymptomatic and microbiologically silent post-infection phase, and granulomatous inflammatory control with eventual granuloma breakdown and tissue necrosis.

Unfortunately, none of the available models can reproduce all of these features. Several animal models have been developed, and while some have become a staple of research, others have fallen out of use. The guinea pig model was developed originally to show that pure cultures of bacilli isolated from patients infected with Mtb produced similar disease when given to healthy guinea pigs (96). This original model is still used today and has been shown to produce disease with low-dose aerosols resulting in the development of human-like granulomas, and animals exhibit BCG-vaccine-mediated protection from cavitary disease (97). The production of granulomatous disease and the demonstration of BCG-mediated protection make guinea pigs an attractive model for drug and vaccine development. Animal-to-animal transmission has also been shown between infected and sentinel animals housed in the same room (98, 99). Recently, a guinea pig model of transmission was reported that optimized airflow and housing conditions for transmission and laid the groundwork to study the requirements of Mtb for effective transmission (100). Previous guinea pig studies have shown that specific cell wall glycolipids are required to induce coughing, but not for pulmonary disease development (101), providing a clear example of the advantages of this animal model. However, the guinea pig model lacks the extensive host genetic tools and probes that limit its broad adoption as a mechanistic model for the pathogenesis of TB, advantages that have enabled mouse models of Mtb infection.

Despite its limitations, the mouse model of aerosol *M. tuberculosis* has emerged as the workhorse of *M. tuberculosis* pathogenesis due to its genetic tractability, abundance of inbred strains, commercial availability, and ease of housing. Aerosol infection of mice with *M. tuberculosis* does not produce the well-structured granulomas that are the signature of human infection, suggesting that mouse models fail to fully capture the immunopathology of human disease. Consistent with this limitation, mouse infection progresses through an innate phase of rapid bacterial replication, followed by immune control and chronic pulmonary infection. Although the murine immune system is incapable of reducing *M. tuberculosis* titers to a state of latency, the chronic infection established by *M. tuberculosis* in mice is a form of immune-bacterial stasis that depends on multiple factors that are now known to be critical in human TB, such as TNF and IFN-γ (102–104). Mouse models have therefore proven useful to elucidate many important aspects of host and Mtb biology. Both B6 and BALB/c mice are commonly used as models for Mtb infection and are classified as highly resistant mouse strains, with mean survival of more than 200 days after high dose I.V. infection compared to other common inbred strain lines (105). Several improvements to the mouse model have been made by modifying infectious dose, using Mtb genetic tools, and harnessing mouse genetics. Ultralow dose infection of C57BL/6J was reported to more closely resemble human disease with the development of well-contained unilateral infection, but unlike in human disease, led to steady increases in Mtb over time and dissemination of bacteria to other organs (106). Several alternative mouse models have also been shown to exhibit immunopathology that resembles human infection, at least in terms of lung cavitation. The highly susceptible C3HeB/FeJ mouse (107) due to deficiency of *sp140* (108) and a mouse model of IL-10 deficiency (CBA/J IL-10^−/−^) (109) develop necrotizing granulomas in the lung. A third granuloma-forming mouse model, utilizing Nos2^−/−^ mice through ear inoculation, allows for the study of granuloma formation (110). Humanized mouse models allow for study of human immune cells within the mouse host (111) and for the study of HIV co-infection (112). Although these individual mouse strains more closely mimic certain elements of human disease, the collaborative cross and diverse outbred mouse populations (113), coupled with systematic infection with transposon mutant libraries, have elucidated a range of host susceptibility and disease phenotypes beyond what could be deduced from single inbred mouse hosts (114–117). This broad range of mouse models all share one deficiency: none of them reproduce paucibacillary disease, i.e., latency. This critical phase of the *M. tuberculosis* infection cycle has been invisible in the existing animal models until recently. To study latency in C57BL/6J mice, a genetic model of Mtb was developed in which conditional depletion of essential genes allows for the establishment of latent infection. This model opens the possibility of studying the immune control of latency in the mouse and the bacterial factors important to maintain latency (118, 119).

Non-human primate (NHP) models utilizing rhesus and cynomolgus macaques most closely parallel human disease with granuloma formation and immune control of disease (120–122), with some animals developing active disease and others remaining latent for months to years post-infection (123). The NHP model also allows study of individual lung lesions utilizing live animal imaging and dissection, providing major insight into lesion heterogeneity and sterilization, insights that would be impossible in other animal models (124, 125). NHP models have also been used extensively in vaccine development and have yielded insights that could lead to improved vaccines for humans (126–130). Although clearly valuable, the wide adoption of NHP models is hampered by the intensity of veterinary husbandry, cost, and need for specialized facilities. Finally, although using *M. marinum* instead of Mtb, the zebrafish model has emerged as an important model and was the subject of a prior review in this series, to which we refer the reader (3).

## *M. TUBERCULOSIS* AS A MODEL FOR DRUG DEVELOPMENT AND DIAGNOSTICS

Despite the massive disease burden of TB, because this burden is concentrated in areas of the world with fewer economic resources, drug and diagnostic development for TB is not a priority for large pharmaceutical companies. This problem is layered on the broader lack of antibiotic development to combat the antimicrobial resistance (AMR) crisis. Out of this necessity, the Mtb research field, supported by governmental and philanthropic sources, has filled this gap and can claim some successes in drug and diagnostic development.

Of course, all therapeutic and diagnostic advances rest on a foundation of basic investigation, and the accumulation of knowledge about Mtb over the past 25 years has been staggering and is the result of many intersecting strands of technical advances, strong public funding, and a highly motivated and interactive mycobacterial research community. Drug targets and pathways elucidated through this work include the critical pathways for respiration, redox balance, and cell wall biosynthesis, showing which bacterial processes are most susceptible to inhibition by new drugs (131). Moreover, well-established laboratory and animal models reproduce key aspects of TB, allowing the testing of new drugs and diagnostics in systems that closely reflect human infection (132). This biological complexity, experimental tractability, and clinical relevance position Mtb as not only a target of therapeutic interest, but also a model organism for understanding and overcoming antimicrobial challenges across diverse bacterial pathogens. We provide some selected examples of the Mtb field serving as a model for therapeutic development.

### Targeting mycobacterial energy metabolism and cell wall biosynthesis

Therapeutic advances targeting energy metabolism and cell wall biosynthesis in Mtb demonstrate how mechanistic insights can be translated into compounds effective against Mtb infection, as shown by Bedaquiline (BDQ), Telacebec (Q203), Pretomanid, and Delamanid (DLM).

BDQ is a diarylquinoline that displays potent *in vitro* activity against both drug-sensitive and drug-resistant strains of Mtb, with a minimum inhibitory concentration of 0.002–0.013 µg/mL. It was discovered in a small molecule screen for compounds active against *M. smegmatis*, emphasizing the importance of microbial model systems for Mtb. It possesses anti-mycobacterial activity by inhibiting ATP synthase, a process critical for Mtb’s active growth as well as its survival in the nonreplicating dormant state (133, 134). The mycobacterial ATP synthase comprises a membrane-embedded, proton-translocating F_0_ domain and a catalytic F_1_ domain. The F_0_ domain includes the a subunit and a ring of nine c subunits (c_9_), which together mediate proton transfer from the intermembrane space to the cytoplasm. Proton flow drives the rotation of the c_9_ ring and the central stalk, thus driving proton translocation to ATP synthesis. BDQ binds at a distinct site located at the interface between the a and c subunits within the F_0_ domain, thereby blocking rotation of the disks and halting the entire process of ATP synthesis (135, 136). Clinically, BDQ has shown that disrupting oxidative phosphorylation can achieve sterilizing activity in chronic infections, providing a foundation for the development of inhibitors that target other components of the Mtb electron transport chain.

Q203, which has completed phase II clinical trials for the treatment of TB (ClinicalTrials.gov identifier NCT03563599), extends the bioenergetic paradigm established by BDQ by targeting the cytochrome *bcc-aa_3_* complex, the heme/copper oxidase of Mtb’s electron transport chain (137). Q203 specifically binds the QcrB subunit of *bcc-aa_3_*, halting electron flow and disrupting the proton gradient required for ATP synthesis. Because Mtb possesses two terminal oxidases, cytochrome *bcc*-*aa*_*3*_ and cytochrome BD, bacteria can compensate for the inhibition of a single branch, whereas simultaneous inhibition of both pathways is synthetically lethal (138). This targeted disruption of respiratory and redox processes emphasizes a broader principle: identifying and exploiting the vulnerabilities in bacterial energy metabolism can guide the development of potent therapies against both active and persistent populations, providing a transferable model for other pathogens. Notable additional successes have been achieved in the TB treatment space, particularly for MDR TB, including the development of Pretomanid (139, 140) and Delamanid (141).

### Translational diagnostics of mycobacteria

Mtb has also served as a model for translating molecular and immunological insights into diagnostic technologies, as exemplified by Xpert MTB/RIF and QuantiFERON. Cepheid Gene Xpert System’s MTB/RIF assay uses quantitative PCR to amplify Mtb DNA directly from a sputum sample. If Mtb DNA is detected, fluorescent probes target *rpoB* to identify mutations associated with rifampicin resistance. Amplification and detection occur within a self-contained cartridge, allowing automated sample processing and real-time monitoring of PCR product accumulation (142). This point-of-care test has revolutionized TB diagnostics and allowed simultaneous rapid detection of the presence of TB and the presence of rifampin resistance, an excellent surrogate of multidrug resistance. As a model of a diagnostic developed initially in academia (143–145), GeneXpert shows how well-defined resistance markers can be converted into rapid diagnostic assays. This success inspired a wave of molecular diagnostics for other pathogens not just in high-income hospital labs but in public health programs around the world, making TB the global proof-of-concept for rapid bacteriological diagnosis (146).

IFN-γ release assays demonstrate how early work on translational TB immunology, coupled with whole genome sequencing, can be translated into a clinically useful diagnostic. The test measures interferon-γ released by CD4^+^ and CD8^+^ T cells (or enumerates numbers of IFN-γ-producing T cells in the case of the T-spot.TB test) when exposed to the Mtb specific antigens ESAT-6, CFP-10, and TB7.7 (147, 148). These proteins are absent from BCG (a fact that emerged out of whole genome sequencing of BCG strains [149]) and from most nontuberculous mycobacteria except *M. kansasii*, *M. szulgai*, and *M. marinum*, enabling specific detection of true past TB infection rather than BCG vaccine response, a major confounder in TST testing (150). The assay illustrates how basic investigation, conducted predominantly in academia, can be harnessed into a new diagnostic.

## SUMMARY

The broad scope of this review inevitably shortchanges many aspects of TB science. By highlighting the broad scope of TB as a model, and the notable progress made by our field in the past ~25 years, we hope to make a broader point. Scientific progress, whether toward a therapeutic or enhanced basic knowledge, requires intersecting strands of investigation, many of which cannot be anticipated. Support for this type of broad scientific inquiry, with the ultimate goal of impacting a disease, requires a broad vision and a sustained funding model from governments and foundations. We hope that the success that we highlight here can be invoked as a model for how a scientific ecosystem can productively evolve to ultimately mitigate one of the world’s most lethal infectious diseases. We hope this ecosystem, and others like it, continues to grow and that the notable progress made in understanding and combating this pathogen can be continued in the coming decades.
